# Postoperative complications and short-term prognosis of laparoscopic pancreaticoduodenectomy vs. open pancreaticoduodenectomy for treating pancreatic ductal adenocarcinoma: a retrospective cohort study

**DOI:** 10.1186/s12957-023-02909-x

**Published:** 2023-01-30

**Authors:** Bin Zhang, Zipeng Xu, Weifang Gu, Junjing Zhou, Neng Tang, Shuo Zhang, Chaobo Chen, Zhongjun Zhang

**Affiliations:** 1 Department of Anesthesiology, The Affiliated Hospital of Jiangnan University, Wuxi, 214122 China; 2 Department of General Surgery, Xishan People’s Hospital of Wuxi City, Wuxi, 214105 China; 3 Department of Laboratory, The Affiliated Hospital of Jiangnan University, Wuxi, 214122 China; 4 Department of Hepatic-Biliary-Pancreatic Surgery, The Affiliated Hospital of Jiangnan University, Wuxi, 214122 China; 5 Department of Hepatic-Biliary-Pancreatic Surgery, The Affiliated Drum Tower Hospital of Nanjing University Medical School, Nanjing, 210008 China; 6 Department of Immunology, Ophthalmology & ORL, Complutense University School of Medicine, 28040 Madrid, Spain

**Keywords:** Pancreaticoduodenectomy, Pancreatic adenocarcinoma, Laparoscopy, China, Retrospective study

## Abstract

**Background:**

Although laparoscopic pancreaticoduodenectomy (LPD) has been accepted worldwide for treating pancreatic ductal adenocarcinoma (PDA), it is a very technical and challenging procedure. Also, it is unclear whether LPD is superior to open pancreaticoduodenectomy (OPD). This study summarized the experience and efficacy of LPD for treating PDA in our medical center.

**Methods:**

This retrospective cohort study included patients with PDA admitted at the Affiliated Hospital of Jiangnan University from October 2019 and January 2021. Patients received either LPD or OPD. Clinical outcomes (operation time, duration of anesthesia, intraoperative hemorrhage), postoperative complications, and short-term outcomes were compared. Cox proportional hazard model and Kaplan-Meier method were used to analyze overall survival (OS) and progression-free survival (PFS).

**Results:**

Among the PDA patients, 101 patients underwent surgical treatment, 4 patients converted from LPD to OPD, and 7 of them received conservative treatment. Forty-six patients were cured of LPD, and 1 of them died shortly after the operation. Moreover, 44 patients received OPD, and there were 2 postoperative deaths. There were significant differences in the location of the operation time, duration of anesthesia, postoperative hemorrhage, abdominal infections, and postoperative pneumonia between the two groups (all *p* < 0.05). Multivariate analysis showed that LPD was an independent factor negatively correlated with the incidence of pneumonia (relative risk (RR) = 0.072, 95%CI: 0.016–0.326, *p* = 0.001) and abdominal infection (RR = 0.182, 95%CI: 0.047–0.709, *p =* 0.014). Also, there were no differences in OS (hazard ratio (HR) = 1.46, 95%CI: 0.60–3.53, *p* = 0.40) and PFS (HR = 1.46, 95%CI: 0.64–3.32, *p* = 0.37) at 12 months between the two groups.

**Conclusions:**

LPD could be efficacy and feasible for managing selected PDA patients. Also, LPD has a better effect in reducing postoperative pneumonia and abdominal infection compared to OPD.

## Introduction

Pancreatic cancer is one of the deadliest malignant diseases originating from the gastrointestinal tract [1]. Most pancreatic cancer patients are asymptomatic in the early stage, thus losing their chance to undergo surgery. Previous studies have reported that only about 15% of patients can receive surgical treatment, and more than 50% of those developed recurrence 2 years after surgery; thus, the 5-year survival rate is low (approximately 20%) [2, 3].

Pancreatic ductal adenocarcinoma (PDA) is a deadly tumor associated with chronic pancreatitis and acinar cell dedifferentiation. Surgical resection combined with adjuvant systemic chemotherapy is the only effective method for improving long-term survival in patients with PDA [4]. However, recent data suggested that differences in age, gender, regional economy, surgical technique, racial disparities, and patient acceptance significantly affect surgical resection in radical cure rates in PDA patients [5]. Corresponding studies have also shown that elderly PDA patients eligible for surgery often refuse an operation [6]. Also, compromised physiological reserve, comorbidities, and the natural history of PDA may deny pancreatic resection in those patients [7].

With the rapid development of minimally invasive surgical techniques, as well as experienced surgeons, together with the improvement of surgical instruments and equipment, patients who were eligible for the traditional OPD approach in the past can now be treated by LPD or even robot-assisted pancreaticoduodenectomy (RAPD) [8, 9]. Since 1994, when the first LPD was performed [10], successful outcomes have been observed in PDA patients who were treated by skilled and experienced surgeons. LPD is currently a feasible option for selected PDA patients at high-volume centers with available experts [1].

Laparoscopic distal pancreatectomy is considered a safe and standard method for treating high-selected left-sided pancreatic tumors [11]. However, the current situation of LPD still has a long way to go in consideration of surgical technology and oncological safety [12]. In addition, there is still controversy about whether LPD or OPD approach should be applied [12, 13]. Previous studies suggested that LPD is not superior to OPD except for causing less intraoperative hemorrhage [14, 15]. In China, LPD is a recommended option for treating pancreatic cancer [1, 16].

Surgeons who implement LPD need to be skilled in laparoscopic techniques and reasonably select appropriate patients to achieve a safe and effective treatment with a negative margin (R0 radical resection) [17, 18]. Otherwise, the positive margin of LPD surgical lesions may seriously affect the survival of PDA patients [19]. Therefore, as suggested, surgeons who perform LPD need to be experienced experts with a clear anatomical hierarchy who have overcome the learning curve of laparoscopic pancreatic surgery [20]. Also, in order for the LPD approach to be successful, PDA patients with resectable lesions should be carefully selected [21].

In China, many medical centers have successively carried out LPD to treat patients with PDA and achieved well feedback during the last 5 years [3, 9, 22]. Furthermore, Zhao et al. [23], together with multicenter joint domestic and foreign experts, proposed an international expert consensus on diagnosing and treating PDA. However, diagnosis and treatment techniques of PDA as well as different concepts may vary in different regions and medical centers, which makes the surgical treatment of PDA uneven. Herein, we retrospectively summarized the treatment experience and efficacy of LPD to PDA in our medical center from October 2019 to January 2021. Simultaneously, we also summarized the experience and effects of PDA treatment in a medical center in eastern China, which may further benefit future treatment.

## Methods

### Study design

This retrospective cohort study included patients with PDA admitted at the Affiliated Hospital of Jiangnan University between October 2019 and January 2021. The study complied with the Declaration of Helsinki and was approved by the Ethics Committee of the Affiliated Hospital of Jiangnan University. The requirement for informed consent was waived.

### Inclusion criteria and exclusion criteria

In this retrospective analysis, PDA patients were screened according to the inclusion criteria and exclusion criteria.

The inclusion criteria were [24] (1) patients who underwent LPD or OPD surgery for PDA tumors; (2) no distant metastasis in preoperative imaging assessment, and the portal vein invasion was less than 180° on preoperative computed tomography (CT) or magnetic resonance imaging (MRI); and (3) patients who were discharged from hospital postoperation and were followed up.

The exclusion criteria were [24] (1) patients who could not tolerate pneumoperitoneum or general anesthesia, (2) patients with severe systemic comorbidities who could not complete the operation or converted from LPD to OPD or died within 30 days after the operation, (3) patients with other abdominal organ resections, (4) patients who received neoadjuvant chemotherapy before operation, (5) patients with benign pancreatic tumors, and (6) patients who could not cooperate with postoperative follow-up.

### Surgical technique

All surgeries were completed by the same surgical team. Surgical procedures of LPD and OPD were performed as previously published [23, 24]. The patients with obstructive jaundice underwent percutaneous transhepatic bile duct drainage (PTCD) and liver protection treatment; the operation was performed when the liver function recovered to Child-Pugh A class [25].

Before the operation, all patients were followed up by a multidisciplinary team, including doctors from the department of general surgery, medical imaging, anesthesiology, oncology, cardiovascular, etc. The selection of LPD or OPD was decided by the patient, while the surgeon decided whether to convert LPD to OPD during the operation according to the focus of the lesion.

### Data collection

Data collection and follow-up were carried out for both LPD and OPD groups during the same period. Collected data included age, gender, weight, height, body mass index (BMI), operation time, duration of anesthesia, intraoperative hemorrhage, American Society of Anesthesiologists (ASA) grade [26], pT, pN, and stage as well as differentiation, studied pathological parameters, and postoperative complications. Both LPD and OPD groups were followed up for 12 months. Overall survival (OS) and progression-free survival (PFS) were statistically calculated.

### Statistical analysis

The data were analyzed using SPSS 22.0 (IBM, Armonk, NY, USA). The continuous data were expressed as means ± standard deviations and analyzed via Student’s *t* test. Categorical data were presented as frequencies and scores and were analyzed using Fisher’s exact test. Non-normally distributed variables were presented as medians with interquartile ranges (IQR) and tested using the Mann-Whitney *U* test. Variables with a *p-*value < 0.05 in the univariable analyses and concerned in the study were included in the multivariable logistic regression (enter method). OS and PFS were analyzed using the Kaplan-Meier and Cox proportional hazard models. A *p-*value < 0.05 was considered statistically significant.

## Results

### Characteristics of the patients

**Fig. 1:**
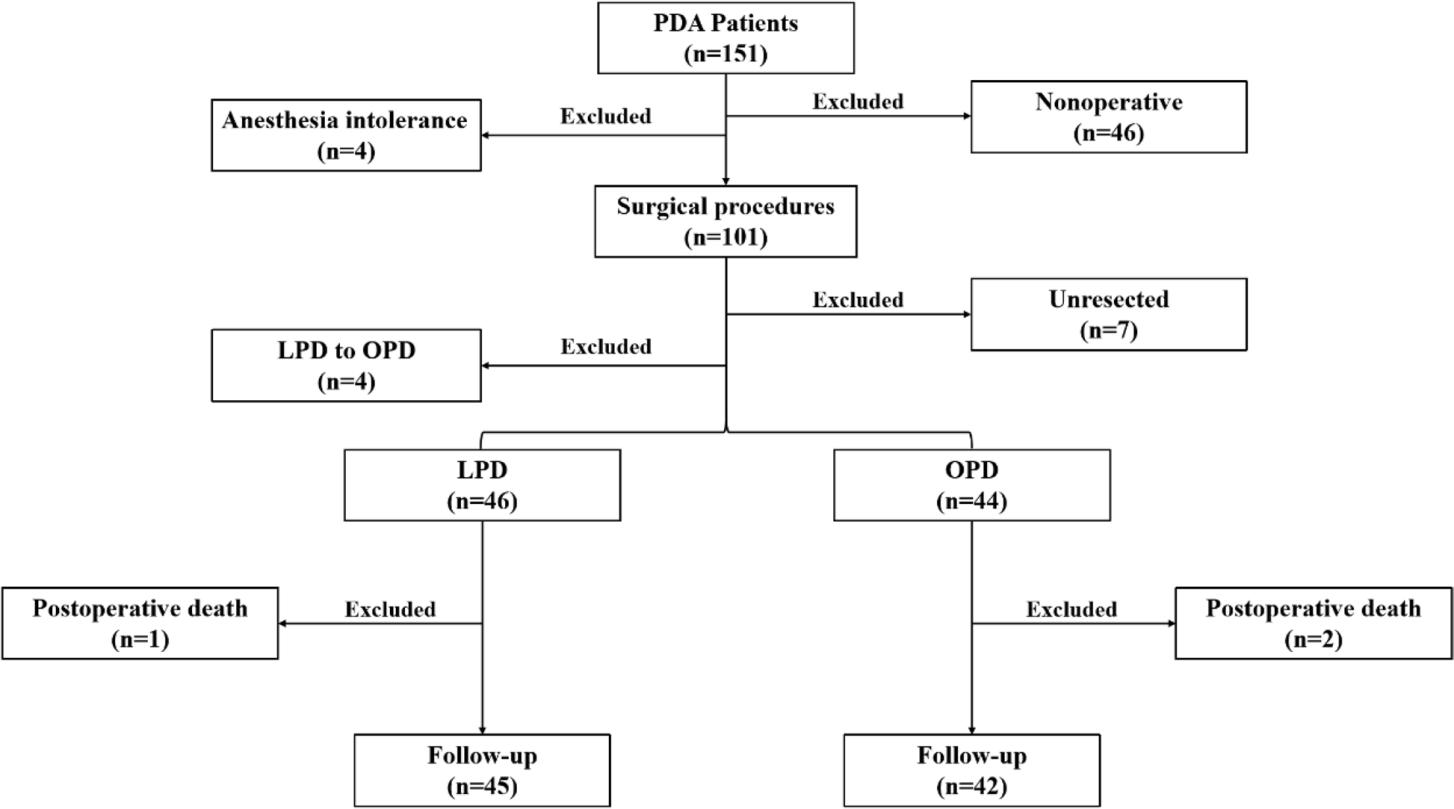
Study flow diagram

A total of 151 patients were diagnosed with PDA at the Affiliated Hospital of Jiangnan University between October 2019 and January 2021. Among these patients, 4 patients with anesthesia intolerance and 46 patients who were not surgically treated were excluded. Among 101 participants who received surgery, 4 patients were excluded for converting from LPD to OPD, and 7 were because the tumor was not fully resected. Forty-six were treated with LPD; among those, 1 patient died within 30 days after the operation. In the OPD group, 44 patients received traditional open surgery and 2 died within 30 days after the operation. Collectively, 45 patients were included in the LPD group and 42 patients in the OPD group. Finally, 45 LPD and 42 OPD patients were successfully followed up (Fig. 1).

The characteristics and clinical features of the patients are shown in Table 1. There were no significant between-group differences in the location of general characteristics, including age, gender, weight, height, BMI, intraoperative hemorrhage, lymph nodes, ASA grade (II to IV), pT, pN, stage, and differentiation (all *p* > 0.05, Table 1). Yet, there were significant differences in the operation time and duration of anesthesia (*p <* 0.001, Table 1).

**Table 1:** Characteristic and clinical features of patients

Items	LPD (***n*** = 45)	OPD (***n*** = 42)	***p-***value
**Age (years)**	63.933 ± 9.034	63.143 ± 9.528	0.696
**Gender (male/female)**	26/19	16/26	0.066
**Weight (kg)**	60.933 ± 12.452	59.000 ± 10.114	0.436
**Height (cm)**	165.444 ± 9.537	163.595 ± 7.416	0.324
**BMI (kg/m**^**2**^**)**	22.110 ± 3.188	21.930 ± 2.545	0.775
**Operation time (h) (IQR)**	8.667 (7.500**–**9.250)	5.208 (4.167**–**6.833)	***< 0.001***
**Duration of anesthesia (h) (IQR)**	9.000 (7.917**–**9.750)	5.583 (4.542**–**7.167)	***< 0.001***
**Intraoperative hemorrhage (ml) (IQR)**	400 (200**–**500)	400 (225**–**500)	0.447
**Lymph nodes**	15.600 ± 3.065	15.095 ± 3.393	0.473
**ASA grade**			
Grade II	29	31	0.454
Grade III	15	11	
Grade IV	1	0	
**pT**			
T1	1	2	0.797
T2	6	6	
T3	38	34	
**pN**			
N0	13	9	0.424
N1	32	33	
**Stage**			
Stage I	3	5	0.449
Stage II	41	37	
Stage III	1	0	
**Differentiation**			
Well	5	7	0.648
Moderate	25	24	
Poor	15	11	

Bold values indicate a *p-*value less than 0.05 is statistically significant

*ASA* American Society of Anesthesiologists

### Postoperative complications

Comparing LPD to the OPD group, there were no significant differences in terms of secondary surgery, bile leakage, pancreatic leakage, chyle leak, and gastrointestinal dysfunction after the operation (Table 2, *p* > 0.05). However, there were 11 postoperative hemorrhages and 3 abdominal infections in the LPD group compared to 3 and 12 in the OPD group, respectively (Table 2, *p* < 0.05). Moreover, 3 postoperative pneumonias were detected in the LPD group compared to 20 in the OPD group (Table 2, *p <* 0.001).

**Table 2:** Postoperative complications of LPD vs. OPD

Items, ***n*** (%)	LPD (***n*** = 45)	OPD (***n*** = 42)	***p-***value
**Secondary surgery**	6 (13.33)	2 (4.76)	0.312
**Bile leakage**	3 (6.67)	8 (19.05)	0.082
**Pancreatic leakage**	6 (13.33)	10 (23.81)	0.208
**Chyle leak**	2 (4.44)	0 (0.00)	0.495
**Hemorrhage**	11 (24.44)	3 (7.14)	***0.028***
**Abdominal infection**	3 (6.67)	12 (28.57)	***0.007***
**Pneumonia**	3 (6.67)	20 (47.62)	***< 0.001***
**Gastrointestinal dysfunction**	2 (4.44)	3 (7.14)	0.937

### Univariable and multivariable analysis for hemorrhage in all patients

Univariate analysis showed that LPD (RR = 4.206, 95%CI: 1.083–16.336, *p =* 0.038), operation time (RR = 1.391, 95%CI: 1.059–1.827, *p =* 0.018), and duration of anesthesia (RR = 1.389, 95%CI: 1.057–1.825, *p =* 0.018) were associated with hemorrhage. Yet, multivariate results suggested that LPD (RR = 2.477, 95%CI: 0.534–11.494, *p =* 0.247), operation time (RR = 2.384, 95%CI: 0.046–122.782, *p =* 0.666), and duration of anesthesia (RR = 0.538, 95%CI: 0.010–28.331, *p =* 0.759) were not independent affecting factors associated with hemorrhage in all patients (Table 3).

**Table 3:** Univariable and multivariable analysis for *hemorrhage* in all patients

	Univariable			Multivariable		
	RR	95%CI	***p-***value	RR	95%CI	***p-***value
**LPD vs. OPD**	***4.206***	***1.083–16.336***	***0.038***	2.477	0.534–11.494	0.247
**Gender**	3.203	0.919–11.161	0.068			
**Age**	0.967	0.910–1.029	0.293			
**BMI**	1.062	0.873–1.292	0.546			
**Operation time**	***1.391***	***1.059–1.827***	***0.018***	2.384	0.046–122.782	0.666
**Duration of anesthesia**	***1.389***	***1.057–1.825***	***0.018***	0.538	0.010–28.331	0.759
**Intraoperative hemorrhage**	1.001	0.998–1.004	0.403			
**ASA grade**	2.215	0.755–6.492	0.147			
**Stage**	0.508	0.099–2.661	0.417			

*RR* relative risk, *CI* confidence interval

### Univariable and multivariable analysis for abdominal infection in all patients

Univariable analysis showed that LPD was negatively correlated with the incidence of abdominal infection. Multivariate analysis indicated that LPD was a protective factor for abdominal infection (RR = 0.182, 95%CI: 0.047–0.709, *p =* 0.014). (Table 4).

**Table 4:** Univariable and multivariable analysis for *abdominal infection* in all patients

	Univariable			Multivariable		
	RR	95%CI	***p-***value	RR	95%CI	***p-***value
**LPD vs. OPD**	***0.179***	***0.046–0.688***	***0.012***	***0.182***	***0.047–0.709***	***0.014***
**Gender**	0.925	0.303–2.820	0.891			
**Age**	1.009	0.950–1.072	0.766			
**BMI**	1.151	0.949–1.397	0.153			
**Operation time**	1.029	0.819–1.293	0.807			
**Duration of anesthesia**	1.019	0.812–1.278	0.874			
**Intraoperative hemorrhage**	1.002	0.999–1.005	0.187	***1.002***	***0.999–1.005***	***0.235***
**ASA grade**	1.059	0.345–3.251	0.921			
**Stage**	0.292	0.064–1.342	0.292			

*RR* relative risk, *CI* confidence interval

### Univariable and multivariable analysis for pneumonia in all patients

Our results showed that the LPD group had a lower rate of pneumonia compared with the OPD group (Table 2). At the same time, the multivariable analysis indicated that LPD was an independent factor in reducing the risk of pneumonia in all patients (RR = 0.072, 95%CI: 0.016–0.326, *p =* 0.001) (Table 5).

**Table 5:** Univariable and multivariable analysis for *pneumonia* in all patients

	Univariable			Multivariable		
	RR	95%CI	***p-***value	RR	95%CI	***p-***value
**LPD vs. OPD**	***0.079***	***0.021–0.294***	***< 0.001***	***0.072***	***0.016–0.326***	***0.001***
**Gender**	2.000	0.757–5.287	0.162			
**Age**	0.993	0.943–1.045	0.780			
**Weight**	1.014	0.973–1.056	0.511			
**Operation time**	0.823	0.670–1.011	0.063	4.933	0.108–225.779	0.413
**Duration of anesthesia**	0.815	0.664–1.001	0.051	0.215	0.005–9.952	0.432
**Intraoperative hemorrhage**	1.001	0.998–1.003	0.677			
**ASA grade**	0.376	0.117–1.209	0.101			
**Stage**	0.520	0.123–2.201	0.375			

*RR* relative risk, *CI* confidence interval

### Survival curve

**Fig. 2:**
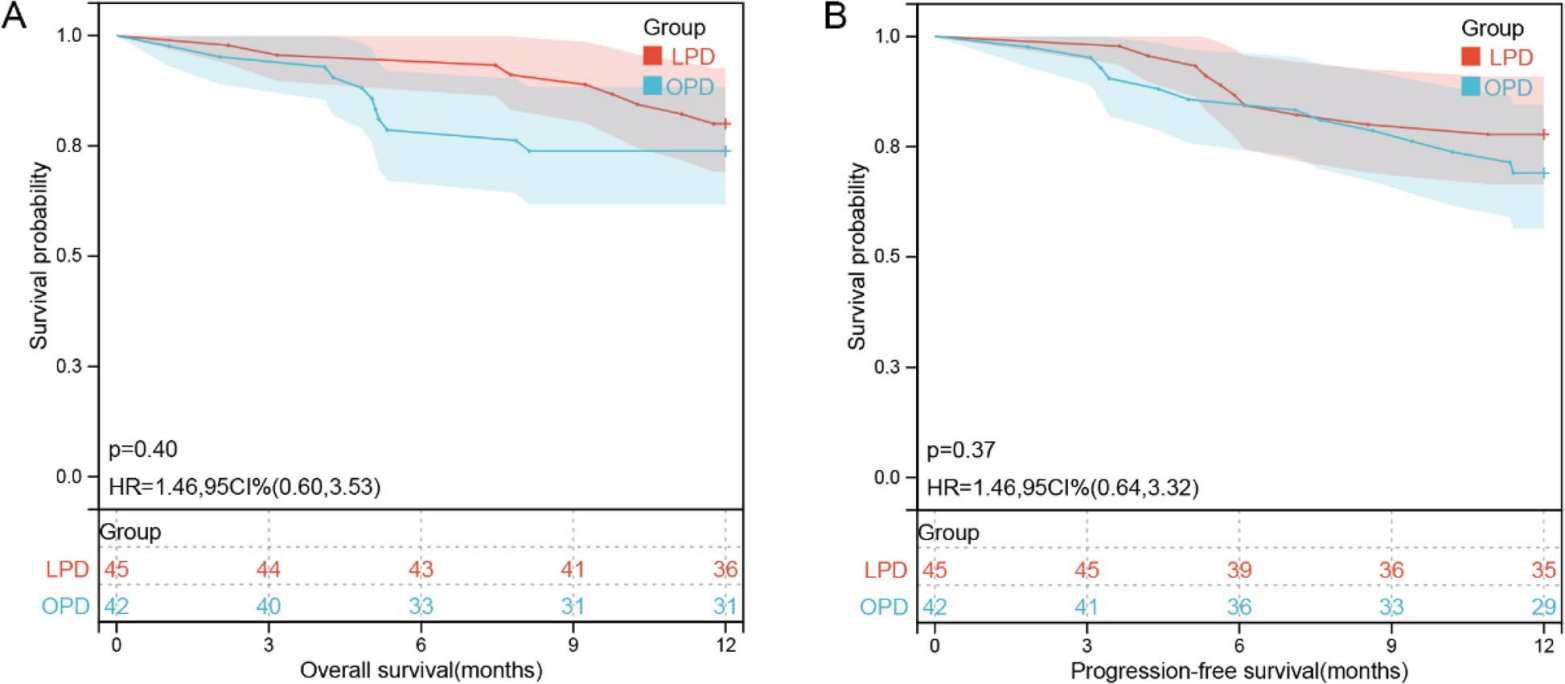
Kaplan-Meier curves for progression-free survival and overall survival in patients who received LPD or OPD. **A** Overall survival. **B** Progression-free survival

All patients received adjuvant chemotherapy as recommended by NCCN clinical practice guidelines [21] after the surgical procedure. Patients were followed up for 12 months postoperation. There was no difference in OS (95%CI: 0.60–3.53, HR (hazard ratio) = 1.46, *p =* 0.40) and PFS (95%CI: 0.64–3.32, HR = 1.46, *p =* 0.37) at 12 months between the LPD and OPD groups (Fig. 2).

## Discussion

Pancreatic cancer is currently the most common gastrointestinal malignancy globally. It is also the fourth leading cause of cancer death in developed countries [27]. Furthermore, experts predicted that pancreatic cancer would soon become the second most common cause of cancer-related deaths [5].

Medical centers are the first-line hospitals for diagnosing and treating PDA in China. This study included 87 PDA patients who received either LPD or OPD treatment. There were significant between-group differences in terms of the operation time, duration of anesthesia, postoperative hemorrhage, abdominal infections, and postoperative pneumonia (Table 2). LPD was independently associated with pneumonia and abdominal infection according to multivariate analysis. The LPD approach was considered safe and feasible in treating selected PDA patients.

Due to the complex operation, which includes a long operation time and high-risk complications, perioperative anesthesia evaluation in patients with PDA is particularly important [19]. The ASA classification of physical condition is the most commonly used score in preoperative evaluation [26]. In this study, ASA scores ranged from grade II to IV, with no statistical differences between the LPD and OPD groups (Table 1). This may also be one of the indications for evaluating the preoperative selection of patients for LPD surgery. In this study, all PDA patients were evaluated according to the inclusion and exclusion criteria described above. One hundred one patients underwent surgical treatment (4 patients converted from LPD to OPD, 7 patients’ conservative treatment); among those, 46 patients were cured of LPD (with 1 postoperative death due to pneumonia), and 44 patients received OPD (with 1 postoperative death caused by abdominal infection and 1 due to liver failure) (Fig. 1). Postoperative mortality rate (less than 30 days) was low in both groups (LPD 1/46 vs. OPD 2/44) (Fig. 1).

Previously, surgical resection combined with systemic chemotherapy was regarded as the only long-term curative option for pancreatic cancer patients [5, 28]. In this study, all patients received adjuvant chemotherapy postoperation as recommended by the NCCN guidelines [21]. However, OS and PFS at 12 months were similar (Fig. 2). Nonetheless, these surviving patients are still followed up in order to facilitate the pooling of later studies on long-term survival.

A recent meta-analysis systematically suggested no significant difference (*p* > 0.05) in the 5-year overall survival compared to LPD with OPD [29]. Another study showed no difference in short-term oncologic outcomes between the LPD and OPD groups but also significantly longer survival (in 3 years, 4 years, and 5 years after the treatment) in the LPD group (*p* < 0.05) [30]. However, another study reported no difference in the length of hospitalization day, R0 radical resection, lymph nodes, and readmission rate in the LPD and OPD groups but a higher postoperative mortality rate (less than 30 days) in the LPD group in the lower volume centers (*p <* 0.05), which increased the focus on the safety of LPD in treating pancreatic cancer [31]. Therefore, it is very important that both LPD and OPD are carried out by experienced pancreatic surgeons, preferably in high-volume centers [31, 32]. In addition, some PDA patients need to be carefully selected when planning the LPD approach, and surgical procedures should also be well designed according to the actual situation of the pancreatic tumor. Some studies have pointed out that in patients with pancreatic cancer, peripheral venous vascular invasion is not a contraindication to LPD [1, 33]. Croome et al. [34] suggested no difference in short-term OS between LPD and OPD combined with vascular resection. In this study, we also found no significant between-group differences in both OS and PFS (Fig. 2). Additionally, there are other modified techniques for reconstruction, such as using the falciform ligament, parietal peritoneum, and teres ligament, which means further potential applications of laparoscopic technique should be studied [1]. For surgeons, laparoscopic combined vascular resection and reconstruction are also feasible; however, they should be performed by an expert with outstanding skills in minimally invasive surgical techniques [35].

The same surgeons performed the preoperative evaluation and surgical operations. Before the operation, all patients were followed up by a multidisciplinary team of doctors. The LPD group had lower rates of abdominal infection and pneumonia (Table 2). However, contrary to previous studies [36–38], more postoperative hemorrhage was found in the LPD group compared to the OPD group in our study. Yet, multivariate analysis indicated that LPD was not an independent risk factor for postoperative hemorrhage. In addition, the effect of laparoscopic pneumoperitoneum, insufflating CO_2_ into the peritoneal cavity results in hypercarbia, acidosis, hemodynamic alterations, and gut ischemia, which may also become the cause [39]. Moreover, fewer postoperative complications with obvious statistical differences in the LPD group were described in the previous study [3]. In this study, however, LPD was independently associated with postoperative pneumonia and was an independent protective factor for abdominal infection (Tables 3, 4, and 5). These findings may have clinical significance for continuing to promote and improve the LPD approach for treating PDA patients. Taking effective postoperative measures to prevent pneumonia and abdominal infection in advance could further reduce LPD postoperative mortality.

In this study, we observed the influence and role of various related factors on LPD and/or OPD approach, and further shared our experience to promote the efficiency of minimally invasive surgery in treating PDA. As these approaches are essential for the treatment of PDA patients, it is necessary for experts to master the professional techniques of pancreatic and laparoscopic minimally invasive surgery and formulate delicate surgical procedures for the patients who meet the indications.

This study has some limitations. More feedback from patients undergoing this procedure is needed. Also, this was a retrospective single-center study with a small sample size. Moreover, no control group was included, and no long-term follow-up was performed. Also, patients were mainly from eastern China, and there was a lack of multi-center and/or regional comparative analysis.

## Conclusion

LPD could be an efficacy and feasible strategy for treating selected PDA patients, yet surgery needs to be performed by expert surgeons who have overcome the learning curve. Also, LPD has a better effect on reducing postoperative pneumonia and abdominal infection compared to OPD. Under the condition of indications, LPD can reduce trauma, present the advantages of minimally invasive surgery, and benefit patients.

## Data Availability

The datasets used and/or analyzed during the current study are available from the corresponding author (Chaobo Chen) upon reasonable request. For any queries, kindly contact bobo19820106@gmail.com.
